# A prospective, randomised trial comparing the quality of recovery between full inhalation anaesthesia and total intravenous anaesthesia post gynaecological surgery

**DOI:** 10.5339/qmj.2026.5

**Published:** 2026-03-03

**Authors:** Purtishil Junghare, Bisman Jeet Kaur Khurana, Hazarika Amarjyoti, Meena Shyam Charan, Naik Naveen, Arora Ashima, Bora Girdhar

**Affiliations:** 1 Department of Anaesthesia & Intensive Care, Postgraduate Institute of Medical Education and Research, Chandigarh, India; 2 Department of Anaesthesia & Intensive Care, Punjab Institute of Liver and Biliary Sciences, Mohali, India; 3 Department of Obstetrics & Gynaecology, Postgraduate Institute of Medical Education and Research, Chandigarh, India; 4 Department of Urology, Postgraduate Institute of Medical Education and Research, Chandigarh, India *Email: amarjyoti28@rediffmail.com

**Keywords:** Anaesthesia, inhalation, intravenous, questionnaires, postoperative

## Abstract

**Background:**

Assessing the quality of recovery (QOR) after open gynaecological surgery done under general anaesthesia (GA) is one of the methods to evaluate anaesthesia outcomes effectively. GA can be administered as full inhalation anaesthesia (FIA) and/or total intravenous anaesthesia (TIVA). Studies have not compared QOR between TIVA and FIA in this cohort. Our study aims to compare the effect of TIVA and FIA on postoperative recovery of patients assessed by the Quality of Recovery-40 (QOR-40) questionnaire.

**Methods:**

One hundred and eight consenting adult patients posted for open gynaecological surgeries under GA were randomly allocated to Sevoflurane-based full inhalation anaesthesia (Group FIA) or propofol-based TIVA (Group TIVA). The primary outcome was comparing the QOR-40 score on the first postoperative day (POD1). Comparing QOR-40 on POD2, extubation time, and Modified Observer’s Assessment of Alertness/Sedation Scale (MOAA/S) score after extubation were the secondary outcomes. Epidural analgesia was provided throughout the study period in both groups.

**Results:**

The mean QOR-40 score in group FIA was not significantly different from that in group TIVA (171.44 ± 7.43 vs. 171.37 ± 7.48; *P* = 0.951). However, within the group, there was a significant reduction in scores in POD1 (*P* < 0.001) and POD2 (*P* < 0.001) from pre-operative values. There was no significant difference in extubation time (*P* = 0.207) and MOAA/S score (*P* = 0.548) between the groups.

**Conclusion:**

Our study found no significant difference in QOR-40 score, extubation time, and MOAA/S score between group FIA and TIVA.

## 1. INTRODUCTION

Anaesthesia outcome is defined as “the occurrence of unexpected complications or mortality during or after anaesthesia that could potentially be attributed to the anaesthetic agents.” For optimising anaesthesia services, it is essential to evaluate anaesthesia outcomes effectively. One way to evaluate outcomes is by assessing the quality of recovery (QOR) of the patients postoperatively using the Quality of Recovery-40 (QOR-40) questionnaire.^1^

## 2. BACKGROUND

Anaesthesia care is broadly divided into general and regional anaesthesia. General anaesthesia (GA) can be further classified as inhalational anaesthesia (IA) and total intravenous anaesthesia (TIVA). TIVA was found to have less immunosuppression and postoperative pain,^2^ whereas with IA, extubation time was shorter and respiratory recovery was faster.^3^ Previous studies have exhibited the advantages and disadvantages of the two anaesthesia modalities.^4^ But there is a lack of studies that compare the QOR between TIVA and IA in patients undergoing open gynaecological surgeries. Hence, we conducted this study to determine which GA technique yields better QOR.

## 3. METHODS

This prospective randomised controlled trial (RCT) was conducted in the operating theatre (OT) of a research hospital between June 2022 and July 2023. The study was approved by the institutional ethics committee and registered with a clinical trial registry before the enrolment of the first patient. Written informed consent was acquired from all participants. This article adheres to the relevant guidelines of the Consolidated Standards of Reporting Trials (CONSORT).

Patients with American Society of Anaesthesiologists -physical status (ASA-PS) I and II, aged 18 years and above up to 60 years, planned for elective open gynaecological surgeries like radical hysterectomy, cystectomy, bilateral salpingo-oophorectomy requiring GA, met the inclusion criteria. Patients with psychiatric disorders, allergies to the anaesthetic, alcohol abuse, nervous system diseases, long-term use of hypnotics or sedatives, anaemia, uncontrolled diabetes and hypertension, severe cardiopulmonary dysfunction, deranged Liver Function Test (LFT), kidney dysfunction on dialysis, and refusal to sign informed consent were excluded.

Patients were randomly allocated using computer-generated random number tables and sequentially labelled opaque sealed envelopes into two groups of 54 patients each: Group FIA (full inhalation anaesthesia), and Group TIVA, in a ratio of 1:1. The anaesthesiologists managing the case were given envelopes before administering anaesthesia. All the participants and preoperative and postoperative follow-up assessors were blinded to the group allocation. Patients fulfilling the inclusion criteria were recruited into the study. Every patient eligible for the study underwent a standard pre-anaesthesia check-up.

### 3.1 Anaesthesia protocol

Patients were kept preoperatively fasting as per standard protocol. After shifting the patient to the OT, ASA standard monitors like pulse oximetry (SpO2), electrocardiogram (ECG), and periodic non-invasive blood pressure were attached along with a neuromuscular monitor, and readings were obtained. Bispectral Index (BIS) sensors were placed on the patient’s forehead before induction of anaesthesia. Intravenous access was secured. For the TIVA group, a separate IV access is established for continuous propofol infusion delivery. Arterial cannulation was performed as needed for continuous arterial blood pressure monitoring. For perioperative analgesia, an epidural catheter was placed preoperatively at the space between L1 and L2 vertebrae appropriate for the proposed surgery using both an 18-G Tuohy needle and an 18-G catheter.

**3.1.1 Group TIVA**

For induction and maintenance of anaesthesia, a target-controlled infusion (TCI) pump was employed for propofol delivery with an initial goal effect-site concentration of 4 to 4.5 μg/mL. The patients were pre-oxygenated with 100% oxygen. Fentanyl at 2 μg/kg was given intravenously, followed by the start of the TCI pump. After the loss of response to verbal command and confirmation of the ability to do bag and mask ventilation, an injury occurred. Vecuronium 0.1 mg/kg was given, and further mask ventilation continued for 3 minutes with 100% oxygen. Anaesthesia was maintained with propofol, targeting an effect-site concentration of 2.5 μg/mL, which was titrated in increments of 0.2 μg/mL based on the BIS index value. After the last skin suture, TIVA was stopped.

**3.1.2 Group FIA**

Anaesthesia was induced by a tidal volume inhalation technique with 6% to 8% sevoflurane as the study inhalation agent, assisted by vecuronium intravenous injection for muscle relaxation with 100% oxygen. Fentanyl at a dose of 2 μg/kg was given. Anaesthesia was maintained with 1.5% to 2.5% sevoflurane, and concentrations were titrated according to the BIS index value. Sevoflurane was stopped after the last skin suture.

After the BIS index is less than 40 and the train of four (TOF) count is 0, endotracheal intubation is done in both groups, and its location is confirmed using end-tidal capnography along with 5-point auscultation. Anaesthesia was maintained within the BIS value of 40 to 60. All patients were ventilated with oxygen and nitrous oxide in a 50:50 ratio, targeting an end-tidal carbon dioxide level of 32 to 35 mmHg. Boluses of vecuronium were given as per the TOF count. An infusion of bupivacaine (0.125%) and fentanyl (2 μg/mL) was used to administer epidural analgesia using an infusion pump. The infusion rate was set at 5 mL/hour in both groups intraoperatively. Rescue analgesia was administered as needed, as assessed by the treating anaesthetist based on increased heart rate and blood pressure in the setting of a Bispectral index suggesting adequate depth of anaesthesia. All patients received an injection. Ondansetron 20 to 30 minutes before surgery. After the end of surgery, the residual neuromuscular block was reversed with an injection. Neostigmine 0.05 mg/kg and glycopyrrolate 0.01 mg/kg. The patient was extubated once the TOF ratio was greater than 90% and the patient had opened their eyes or obeyed the spoken command. Then, patients were shifted to the postanaesthetic care unit (PACU) for further monitoring. The patients were discharged to the post-recovery ward when the Aldrete score was 9 or more. Epidural analgesia was continued postoperatively, and rescue analgesic agents, paracetamol and tramadol, were given to relieve pain when the visual analogue scale (VAS) was >3.

The study participants were visited on the first and second postoperative days, and their recovery status was assessed using the QOR-40 questionnaire, which evaluates five dimensions of recovery, including 12 items evaluating physical comfort, nine items evaluating emotional state, five items evaluating physical independence, seven items evaluating psychological support, and seven items evaluating pain. Each item was rated on a five-point Likert scale: none of the time, some of the time, usually, most of the time, and all of the time.^5–7^ The total score on the QOR-40 ranges from 40 (poorest QOR) to 200 (the best QOR). The QOR-40 questionnaire was used at three time points: T1—on the day before surgery. T2-Postoperative Day-1 (POD1), and T3-Postoperative Day-2 (POD2). The QOR-40 has been used in previous studies involving gynaecological surgery.^8^

The QOR-40 score on POD1 was our primary outcome. Extubation time, modified observer’s assessment of alertness/sedation scale (MOAA/S) score after extubation, and adverse reactions (if any) were recorded as secondary outcomes.

The sample size calculation was done according to the primary outcome (QOR-40 score). In a study by Lee et al., the mean QOR-40 score of IA was 174 (standard deviation [SD]: 17).^6^ Anything above this cutoff was considered a good recovery. There is no literature comparing QOR-40 between the modalities of anaesthesia under study. Therefore, based on the assumption that a 10-point difference represents a 15% improvement in the QOR, a total of 98 patients were required to achieve 90% power with an α of 0.05. Accounting for a 10% dropout rate, each group needed 54 patients in this trial.

The data were tested for normal distribution using the Shapiro-Wilk test. Normally distributed data were expressed as mean and SD, and the unpaired *t*-test was used for intergroup comparisons and the paired *t*-test for intragroup comparisons. Non-normally distributed quantitative and ordinal data were calculated using the Mann–Whitney *U* test. Categorical variables were analysed using the chi-square test. Data collected at multiple points in time were measured using analysis of variance (ANOVA), and post hoc pairwise comparisons were Bonferroni corrected. A two-tailed *P*-value of 0.05 was considered statistically significant. All analysis was performed using R, version 4.2.2.

## 4. RESULTS

**Figure 1.**
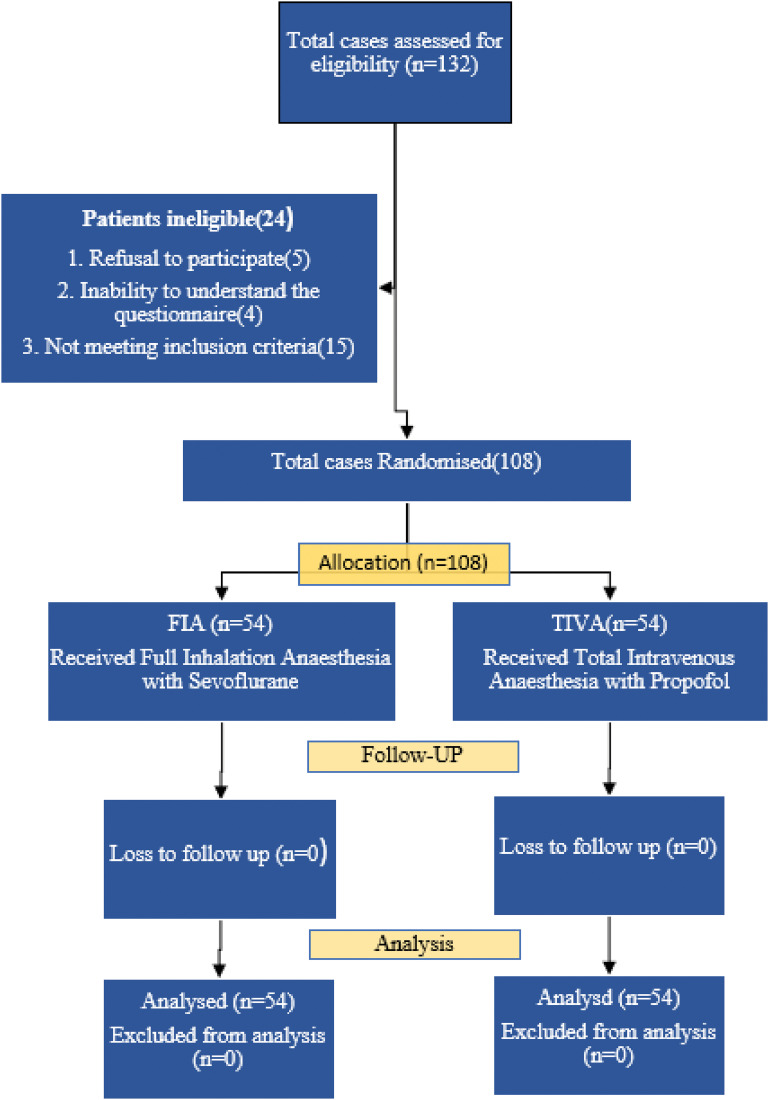
CONSORT diagram showing the patient recruitment, randomisation, and analysis.

One hundred and eight patients were randomly assigned and distributed equally into two groups of 54 patients each. All the patients received the allocated intervention (Figure 1). The two groups were comparable in terms of age, ASA grading, diagnosis, and type of surgery performed (Table 1).

**Figure 2.**
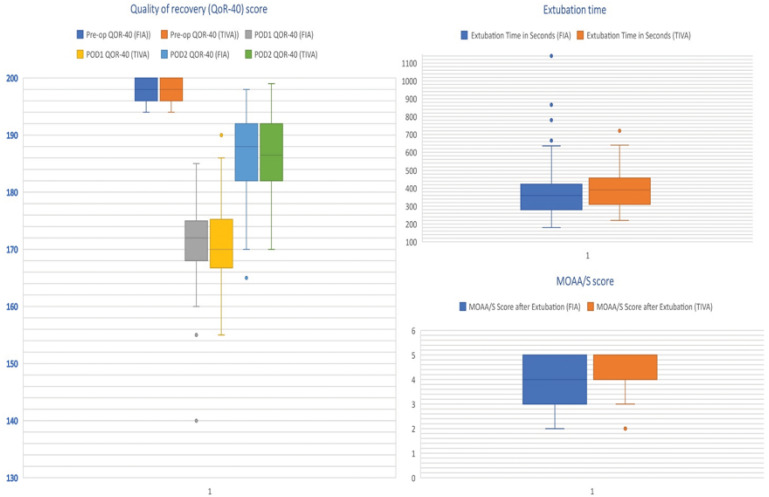
Box and whisker plot presentation of QoR-40 score, extubation time, and MOAA/s score of Group FIA and Group TIVA.

The mean QOR-40 score on POD1 for group FIA was 171.54, and for group TIVA was 171.37. with no statistically significant difference (*P* value, −0.561; Table 2). The difference in mean ΔQOR1 between the groups was also not statistically significant (*P* value, −0.515). We observed a statistically significant reduction in the score at POD1 within the group (Δ QOR1, 26.3 ± 7.2; *P*-value 0.00 and 26.4 ± 7.8, 0.00 in group FIA and TIVA, respectively). On POD2, the mean score improved in both groups, but the difference was not statistically significant (*P* = −0.882). However, this score improvement (ΔQOR3) was significant within the group when compared to POD1 (*P*-value, 0.00; Figure 2). The difference in median extubation time and MOAA/S score was not statistically significant between the groups (Table 2). There were no complications in the groups.

## 5. DISCUSSION

In the realm of gynaecological pathology requiring surgery, pelvic exenteration is the most radical treatment option and is commonly performed using an open approach.^9^ Due to the complexity and invasiveness of these procedures, the QOR becomes critically important in achieving favourable surgical outcomes. Anaesthesia, too, can affect the quality of postoperative recovery. To date, evidence is limited in supporting or disputing the advantage of one aesthetic technique over another.

Our primary outcome was the QOR-40 score assessed at POD1, which was similar in both groups. A comparable QOR-40 score was reported on patients posted for total laparoscopic hysterectomy,^10^ and in a meta-analysis that included laparoscopic gynaecology surgery.^11^ However, a study on laparoscopic donor nephrectomy found significantly better QOR-40 scores in favour of the TIVA group.^7^ Similarly, Lee et al., in their study, found significantly better QOR-40 scores in the TIVA group.^6,10^ The difference in the results of these studies from those of ours is probably due to the different methodology: These studies used propofol during induction for both groups and used either TIVA or FIA for anaesthesia maintenance. Our study used the same anaesthetic technique for both induction and maintenance, targeting the BIS index. Guidelines have recommended using BIS monitoring whenever TIVA is used with a TCI pump.^12^ Also, we provided epidural analgesia starting intraoperatively, continuing it into the postoperative period, in contrast to the use of PCA with morphine and an intermittent bolus of fentanyl for analgesia.

In our study, we found a significant decrease in the mean QOR-40 score on the POD1 in both groups. These can be attributable to various mechanisms, like an inflammatory response, postoperative pain, postoperative nausea and vomiting, and the patient’s perception of physical and psychological well-being post-surgery.^13^ Studies have also reported a significant decrease in QOR-40 score on POD1 compared to preoperative scores.^10,14^

Our mean scores in both groups on POD1 were around 171 (SD-7). This value falls within the cut-off range of good recovery.^6^ Myles et al., categorised the QOR-40 score according to the extent of surgeries into minor, intermediate, and major extents of surgery, and accordingly, its cut-off values were 182 ± 16, 177 ± 14, and 173 ± 14, respectively.^14^ The surgeries that were performed in our study fall under the major category.

There was an improvement in the QOR-40 score on POD2 compared to POD1. Another study also found an improvement in the QOR-40 score on POD2.^13,15^ A possible explanation could be due to a gradual decrease in the inflammatory process as the body recovers from the tissue damage after surgery, which is reflected in improvements in pain, emotional, and physical dependence scores. Also, providing continuous epidural analgesia helps reduce the use of opioid supplements, leading to faster recovery. Moreover, ambulation after extensive surgeries, on average carried out on POD2, invariably improves the psychological factors of a patient.^16^

Extubation time, one of the secondary outcomes, was not statistically different between the groups, although FIA had a shorter time. Studies have reported comparable extubation times between the groups.^15–18^ A systematic review observed that extubation time was significantly quicker for volatile anaesthesia.^17,19^ These similar findings in our study can be attributable to the timely reversal of muscle relaxation and BIS index-guided anaesthetic delivery. We also observed a median MOAA/S score of 4 in both groups, indicating minimal residual effects of the anaesthesia drugs, with other studies reporting similar findings of Niu et al.^9^ Using a BIS monitor and provision for epidural analgesia contributed to a smoother emergence from anaesthesia with a lower incidence of emergence agitation. The significance of prompt emergence from anaesthesia extends beyond immediate postoperative factors like facilitating swift transfer from the operating room (OR), reducing the workload on recovery room staff, and minimising costs.^20,21^

The strength of our study is that this is the first-of-its-kind RCT comparing TIVA and FIA delivered targeting the BIS index based on postoperative QOR. The use of epidural analgesia conforms to analgesic modalities for early recovery. Also, the assessor of our primary outcome was blinded. However, our study still had a few limitations. First, we didn’t include long-term postoperative follow-up, which is an important metric for assessing recovery. Secondly, the surgeries were performed by multiple surgeons. This can impact the generality of any surgical procedure and the overall postoperative recovery. Also, there can be individual variation in the perception of well-being. A cost analysis could have given a clear idea about the general applicability and feasibility of both anaesthesia regimens. Another limitation of the current study was the absence of biochemical markers like inflammatory cytokines and cortisol levels, which are known to affect the recovery of patients in the postoperative period.

## 6. CONCLUSION

The postoperative recovery score of the patients measured by the QOR-40 questionnaire at POD1 between sevoflurane-based FIA and propofol-based TIVA had no significant difference. Extubation time and MOAA/S were also comparable between the two groups. These show that both techniques of GA have similar effects on the postoperative recovery profiles of the patients. To have a more prudent assessment of which form of GA is better, conducting a multicentre trial with a large sample size may provide a valuable conclusion.

## CONFLICT OF INTEREST

The author(s) declared no potential conflicts of interest with respect to the research, authorship, and/or publication of this article.

## AUTHOR CONTRIBUTIONS

PJ: Investigation, Project Administration, and Data Curation. BK: Writing – Original Draft Preparation and Review. AH: Conceptualization, Methodology, and Project Administration. NN: Validation and Formal Analysis. SM: Data Curation. AA: Supervision. GB: Data Collection.

## Figures and Tables

**Table 1.** Showing patient characteristics, surgical, and anaesthesia parameters.

Particulars	Total	Group FIA	Group TIVA	Abs diff (95% CI)	*P*-value*
Age (mean ± SD)	44.1 ± 10.98	44.4 ± 11.10	43.9 ± 10.80	0.3 (-0.82 to 2.45)	0.827^a^
ASA status (n)					0.083^b^
I	54	32	22		
II	54	20	34		
**Diagnosis**					
CA cervix	7	3	4		0.182^b^
CA endometrium	24	10	14		
Ovarian mass	57	26	31		
Fibroid uterus	20	15	5		
Total	108	54	54		
**Name of surgery**					
Debulking surgery	15	6	9		0.731^b^
Myomectomy	19	14	5		
Staging laparotomy + BSO	37	21	16		
TAH + BSO	37	13	24		
Total	108	54	54		
Intraoperative injection of fentanyl (n)	77	36	41		
Cumulative dose (µg)	32.21 ± 14.66	33.89 ± 16.95	30.73 ± 12.33	3.16 (-3.52 to 9.83)	0.349^a^
Duration of surgery (in minutes)	154.86 ± 58.36	161.48 ± 61.69	148.24 ± 54.59	13.21 (-8.99 to 35.47)	0.240^a^
Duration of anaesthesia (in minutes)	179.91 ± 62.57	185.56 ± 66.14	174.26 ± 58.87	11.30 (-12.59 to 35.18)	0.350^a^
PACU stay (in minutes)	117.64 ± 20.92	116.67 ± 21.46	118.61 ± 20.52	−1.94 (-9.95 to 6.07)	0.631^a^

N: Number of patients; PACU: Postanaesthetic care unit; ASA: American Society of Anesthesiologists grade; TAH: Total abdominal hysterectomy; BSO: Bilateral salpingo-oophorectomy; QOR: Quality of recovery.

^a^Unpaired *t*-test.

^b^Chi-square test.

*P < 0.05 is statistically significant.

**Table 2.** Compares the primary and secondary outcomes, intraoperative fluid volume, and surgical blood loss, use and dose of inotropes/vasopressors, and rescue analgesia.

QoR-40	Total	FIA	TIVA	Abs diff (95% CI)	*P*-value^*^
**Preoperative**	197.84 ± 1.81	197.87 ± 1.80	197.81 ± 1.83	0.06 (-0.64 to 0.75)	>0.9^a^
** POD1**	171.41 ± 7.42	171.44 ± 7.43	171.37 ± 7.48	0.07 (-2.77 to 2.92)	>0.9^a^
** POD2**	187.65 ± 6.92	186.59 ± 7.10	189.96 ± 6.68	−3.37 (-6.00 to −0.739)	0.036^a^
**DQoR1 (Pre-op- POD1)**	26.35 ± 7.55	26.32 ± 7.25	26.41 ± 7.81	−0.09 (-2.96 to 2.78)	>0.9^a^
***P*-value^b^**		<0.001	<0.001		
**Abs diff, (95 CI)**		26.43 (24.36 to 28.49)	26.36 (24.36 to 28.51)		
**DQoR2 (POD1-POD2)**	16.35 ± 0.44	15.11 ± 0.28	17.53 ± 0.78	−2.42 ( −2.64 to −2.19)	0.0002^a^
***P*-value^b^**		<0.001	<0.001		
**Abs diff, (95 CI)**		−15.15 (-17.92 to −12.37)	−18.89 (-21.29 to −15.88)		
**Incidence of PONV needing treatment (%; n)**					0.631^c^
** POD1**	38	25	13		
** POD2**	10	8	2		
**Extubation time (seconds; median, IQ)**	367.5 (300–432.5)	360 (280–420)	391 (312–456)	22.7 (12 to 43)	0.207^d^
**Median MOAA/S score (median [IQ])**	4 (4–5)	4 (3–5)	4 (4–5)	0.5 (1 to 1.5)	0.548^d^
** 2**		1	1		0.891^c^
** 3**		13	9		
** 4**		26	29		
** 5**		14	15		
**Total**		**54**	**54**		
**Heart rate (beats/min)^a^**					
** Baseline**	75.43 ± 4.68	76.93 ± 4.69	74.86 ± 4.71	1.07 ( −0.72 to 2.86)	0.239
** 15 minutes**	77.85 ± 5.55	79.52 ± 4.71	76.19 ± 5.87	3.33 (1.30 to 5.36)	0.001
** 30 minutes**	78.88 ± 4.47	79.52 ± 4.71	78.01 ± 4.26	2.50 (0.79 to 4.22)	0.004
** 60 minutes**	77.72 ± 5.11	76.19 ± 5.87	78.52 ± 4.68	−2.33 (-4.35 to −0.30)	0.024
** 1.5 hours**	77.09 ± 4.13	77.02 ± 3.76	77.17 ± 4.46	−0.15 (-1.72 to 1.43)	0.852
** 2 hours**	77.34 ± 3.92	77.21 ± 4.66	77.43 ± 3.75	−0.22 ( −1.83 to 1.39)	0.787
** 2.5 hours**	79.59 ± 4.31	79.73 ± 4.38 (51)	79.56 ± 4.23 (50)	0.17 ( −1.53 to 1.87)	0.843
** 3 hours**	77.87 ± 6.25	77.68 ± 7.68 (31)	78.06 ± 3.85 (32)	−0.38 ( −3.42 to 2.66)	0.803
**Mean arterial pressure^a^**					
** Baseline**	80.87 ± 5.88	80.50 ± 6.35	81.11 ± 5.21	−0.61 (-2.82 to 1.60)	0.586
** 15 minutes**	75.02 ± 5.12	75.48 ± 5.68	74.79 ± 4.95	0.69 ( −1.34 to 2.72)	0.690
** 30 minutes**	73.91 ± 5.81	74.57 ± 5.68	73.56 ± 6.08	1.01 (-1.23 to 3.25)	0.374
** 60 minutes**	74.43 ± 6.17	74.42 ± 6.12	73.46 ± 6.30	0.96 (-1.40 to 3.32)	0.960
** 1.5 hours**	72.34 ± 6.61	72.67 ± 6.39	71.85 ± 7.01	0.82 (-1.73 to 3.37)	0.820
** 2 hours**	71.88 ± 6.72	71.83 ± 6.41	72.02 ± 7.12	−0.19 (-2.77 to 2.39)	0.884
** 2.5 hours**	72.61 ± 5.98	72.59 ± 5.72 (51)	72.74 ± 6.11 (50)	−0.15 (-2.48 to 2.18)	0.898
** 3 hours**	72.01 ± 5.55	71.72 ± 5.82 (31)	72.25 ± 5.37 (32)	−0.53 (-3.34 to 2.28)	0.708
**Analgesia**					
** Cumulative dose**					
** Paracetamol (gm)**		3 ± 0.55	2.96 ± 0.58	0.04 (-0.18 to 0.25)	0.734^a^
** Tramadol (mg; n)**		150 ± 51.30 (20)	111.03 ± 51.01 (29)	−38.97 (-68.86 to −9.07)	0.011^a^

^a^Unpaired t-test.

^b^Paired t-test.

^c^Chi-square test.

^d^Mann-Whitney U test.

*P < 0.05 is statistically significant.

n: Number of patients; PACU: Post-anaesthesia care unit; CI: Confidence interval; IQ: Interquartile; QoR: Quality of recovery; PONV: Postoperative nausea and vomiting; *P* values are Bonferroni adjusted in case of repeated measures.
